# The experience of taking methotrexate for juvenile idiopathic arthritis: results of a cross-sectional survey with children and young people

**DOI:** 10.1186/s12969-015-0052-6

**Published:** 2015-12-12

**Authors:** Kathleen Mulligan, Lucy R Wedderburn, Stanton Newman

**Affiliations:** School of Health Sciences, City University London, Northampton Square, London, EC1V 0HB UK; East London NHS Foundation Trust, London, UK; Infection, Inflammation and Rheumatology Section, UCL Institute of Child Health and Great Ormond Street Hospital NHS Foundation Trust, London, UK

**Keywords:** Juvenile idiopathic arthritis, Methotrexate, Quality of life

## Abstract

**Background:**

Children and young people (CYP) with juvenile idiopathic arthritis (JIA) are known to have impaired health-related quality of life (HRQoL), which is improved significantly for many by treatment with methotrexate (MTX). However, a significant proportion of CYP experience difficulties in taking MTX, which may reduce its potential benefits for HRQoL. The aim of this research was to examine how CYP with JIA perceive MTX treatment and how this relates to HRQoL.

**Methods:**

CYP aged 8–16 years taking MTX for JIA completed an adapted Parent Adherence Report Questionnaire, which contains 100 mm visual analogue scales, to assess difficulty taking MTX, adherence, frequency of negative reactions and helpfulness of MTX. They also completed the Pediatric Quality of Life Inventory (PedsQL) Generic and Rheumatology scales. We collected data on age, gender, JIA course, disease duration, MTX duration of use, route and dose. Number of inflamed and limited joints were indicators of disease severity.

**Results:**

One hundred sixteen CYP participated. Most considered MTX helpful (median 87; interquartile range (IQR) 50.75–98) and reported adherence was high (median 98; IQR 90–100). There was greater variability on scores for difficulty (median 22; IQR 2–69) and frequency of negative reactions (median 14.5; IQR 1.25–80). Mean (S.D.) scores on the PedsQL Physical and Psychosocial subscales were 71.63 (24.02) and 71.78 (19.59) respectively, indicating poorer HRQoL than that reported by healthy children. After controlling for demographic and disease variables, poorer physical HRQoL was significantly accounted for by greater difficulty in taking MTX. Poorer psychosocial HRQoL was significantly accounted for by subcutaneous MTX administration, a lower rating of MTX helpfulness and a greater reported difficulty in taking MTX.

**Conclusions:**

Taking MTX for JIA was viewed as helpful by most CYP but HRQoL was poorer in those who reported greater difficulty in taking MTX.

## Background

Children and young people (CYP) with juvenile idiopathic arthritis (JIA) are known to have impaired health-related quality of life (HRQoL), particularly on measures of the physical domain [1, 2]. Although this is improved significantly for many CYP by treatment with methotrexate (MTX) [3] and biologic therapies [4], HRQoL can remain suboptimal [5]. Higher pain scores and poorer physical function are important predictors of poorer HRQoL in JIA [6] but variability in HRQoL is not explained purely by these factors [7]. For example, Seid et al. [5] found that many CYP with no or mild symptoms still report impaired HRQoL.

A factor that may influence HRQoL in JIA is how CYP experience their treatment. Although MTX has been found to improve HRQoL in JIA [3], CYP may experience side effects such as nausea and vomiting and procedural distress [8, 9]. Approximately half of CYP who take MTX for JIA are reported to experience difficulties. We have previously reported proxy data from mothers of CYP with JIA which found that feeling sick after taking MTX and anxiety about injections were related to poorer HRQoL [9]. Such proxy reports are essential in child health, particularly in relation to younger children, but given the differences found between patient and proxy reports on other measures [10–12], CYP’s own reports of their experiences of taking medication for JIA are also needed.

We are aware of two studies in JIA that have examined the relationship between CYP’s views about their treatment and their HRQoL. Seid et al. [7] found a relationship between greater self-reported treatment problems assessed with the PedsQL Rheumatology Module [2] and poorer physical and psychosocial HRQoL. A study which examined HRQoL in JIA using self-reports from CYP aged 8 years and over, identified ‘subjective burden of medication use’ as a predictor of psychosocial HRQoL in JIA [13]. Neither of these studies asked specifically about MTX and we are not aware of any research that has examined CYP’s own reports of taking MTX and how this impacts on their HRQoL. The aim of this study was to examine how CYP with JIA perceive their MTX treatment and how this relates to their HRQoL.

## Methods

### Design

A cross-sectional design was used. Data were collected as part of the Childhood Arthritis Response to Medication Study (CHARMS), which investigates factors that influence response to MTX or anti-TNF treatment for JIA. This study examines genetic, immunological and psychological aspects of response to medication and recruits CYP who are about to start taking methotrexate (MTX) or anti-TNF, are taking MTX at the time of recruitment or have taken MTX in the past. The study methodology has been described in detail elsewhere [9].

### Participants

Participants were recruited from Great Ormond Street Hospital for Children and the Adolescent Rheumatology service at University College Hospital, London, UK between May 2006 and May 2008. Patients were eligible to take part in the CHARMS study if they had a diagnosis of JIA defined by International League of Associations for Rheumatology (ILAR) criteria [14]. Although CHARMS recruits patients of any age, only patients aged 8 years and over completed questionnaires about their experience of MTX. Not all CYP in the study were still taking MTX at the time the study questionnaires were completed. As some CYP may have ceased taking MTX because they were well but others may have ceased due to intolerance, this analysis is restricted to those CYP who were taking MTX at the time of questionnaire completion to help ensure a more homogeneous sample.

### Procedures

Parents were approached to take part in the CHARMS study at a routine out-patient appointment. Written informed consent was obtained from at least one parent and age-appropriate written assent was obtained from the patient. CYP completed the questionnaires described below during waiting time in the clinic. They were given the option to complete the questionnaires independently or for the researcher to read through the questions for them. The researcher was also available to answer any queries from CYP who chose to complete the questionnaires independently. Parents did not assist CYP with questionnaire completion.

### Ethics, consent and permissions

The study had full ethical approval from the Institute of Child Health/GOSH Local Research Ethics Committee, reference 05/Q0508/95. All participants gave full, informed written consent (parental consent and age appropriate child/young person assent). The study conforms to the principles outlined in the Declaration of Helsinki.

### Measures

Participants completed the following questionnaires:

- Views about MTX were assessed by adapting the Parent Adherence Report Questionnaire (PARQ) [15] so that the questions were addressed to the CYP instead of the parent. CYP indicated on a 100 mm horizontal VAS i) their level of difficulty in taking MTX with endpoints very easy/very hard; ii) how often they take MTX as prescribed with endpoints never/always; iii) negative reactions such as crying in response to taking MTX with endpoints never/always and iv) their opinion of the helpfulness of MTX for their arthritis with endpoints not helpful/very helpful. A mean of questions i) – iii) is calculated to provide an ‘ability to take’ score. Higher scores represent greater perceived ability to take and greater perceived helpfulness.
- HRQoL was assessed using the Pediatric Quality of Life Inventory (PedsQL) Generic and Rheumatology scales [2]. The generic scale provides physical and psychosocial composite scores. The rheumatology scale has 5 subscales: pain and hurt; daily activities; treatment; worry; communication. The composite and subscale scores are each transformed to 0–100 scores as specified by Varni et al. (2002) [2], where a higher score represents better HRQoL.
- We also collected data on the child’s age, gender, JIA type according to ILAR criteria [14] (systemic, oligoarticular persistent, oligoarticular extended, polyarticular RF-, polyarticular RF+, psoriatic, enthesitis-related arthritis (ERA), undifferentiated) disease duration, MTX duration of use, route and dose. The number of inflamed/active and limited joints was recorded as indicators of current disease activity.

### Statistical analysis

Statistical analysis was performed in IBM SPSS Statistics 22.

Medians and interquartile ranges (IQR) were calculated for scores on the PARQ. To examine the hypothesis that CYP’s views of MTX would account for some of the variance in HRQoL measured with the PedsQL, the relationship between variables was examined initially by correlations (Pearson r correlations for continuous variables, Spearman’s rho (r_s_) for ordinal variables). In the case of categorical independent variables (e.g. gender), differences in HRQoL between categories were examined by *t*-test or analysis of variance (ANOVA), as applicable. As expected, we recruited small numbers of CYP with the lower prevalence JIA types (psoriatic = 6; ERA = 7; undifferentiated = 2); therefore we classified participants into whether they had an oligoarticular or polyarticular course, that is the number of joints that had been involved up to the time of the study (4 or less, more than 4 respectively).

To examine which variables accounted for most variance in HRQoL, all significant variables identified from the univariate analyses were included in hierarchical multiple regressions using enter method and a level of *p* < .05 as an entry criterion.

Two regression analyses were performed, one for the Physical and one for the Psychosocial summary scales of the Generic PedsQL. The independent variables were entered into the regression in blocks in the following order: 1. Demographic variables; 2. Disease variables; 3. MTX-related variables. This order was used because it enables examination to be made as to whether experience of MTX added to the explanation of quality of life once disease severity had been taken into account.

## Results

One hundred sixteen CYP who were taking MTX at the time of study recruitment completed the study questionnaires. Sample characteristics are shown in Table 1. As expected in JIA, the majority of CYP were female, and for most, their JIA had taken a polyarticular course, affecting 5 or more joints. A small majority of CYP (54.3 %) were taking MTX subcutaneously at time of assessment.

**Table 1:** Sample characteristics

*n*	116
Gender, n (%) female	77 (66.4)
Age in years when questionnaire data completed, mean (S.D.)	11.9 (2.2)
JIA course, n (%)	
*systemic*	14 (12.1)
*oligoarticular*	11 (9.5)
*polyarticular*	91 (78.4)
Disease duration in years, mean (S.D.)	5.5 (3.4)
Current disease severity, median, range, (IQR)	
Number of active joints (data for *n* = 111)	0, 0-10, 0-2
Number of limited joints (data for *n* = 108)	1, 0–32, (0–3)
Duration of MTX use in years, median (IQR)	2 (1–5)
MTX current route	
*Oral, n (%)*	53 (45.7)
*Subcutaneous, n (%)*	63 (54.3)
Current MTX dose in mg/m^2^/week, median (IQR)	15 (12.5 – 20.0)

**Fig. 1:**
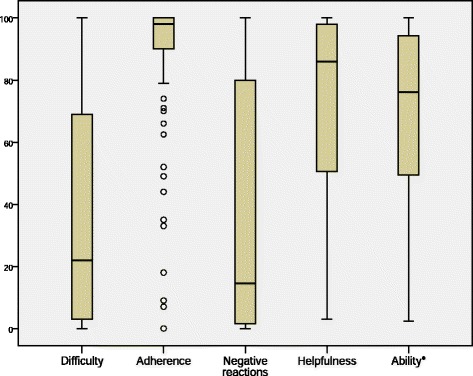
Shows boxplots of scores on the adapted PARQ measure

CYP’s views about MTX are shown in Fig. 1, which reports the median and inter quartile range (IQR) scores on the PARQ. Self-reported adherence was very high among most CYP, with a median (IQR) of 98 (90–100) on the 100 mm scale, however 20 (17.4 %) scored below 80, and of these, 9 (7.8 %) scored below 50. Scores on the other items of the PARQ showed greater variability. A quarter of CYP scored 69 or above on the 100 mm scale for level of difficulty in taking MTX and 80 or above on the 100 mm scale for frequency of negative reactions to MTX. Most CYP rated MTX as helpful with half scoring 87 or above on level of helpfulness however a quarter scored on or below the midpoint of the scale.

Scores on the PedsQL Generic Scale and Rheumatology Module are shown in Table 2. Mean scores on the Rheumatology Module and Physical and Psychosocial subscales were similar to those recorded by the scale's developers in children with JIA [2]. Scores were poorer than those reported by a healthy UK sample, aged 8–18 years, of 88.51 (11.62) and 81.84 (13.21) respectively [16].

**Table 2:** Participant scores on the Generic Core Scales and Rheumatology Module of the Pediatric Quality of Life Inventory (PedsQL)

PedsQL, Generic Scale, mean (S.D.)^a^	
Physical	71.63 (24.02)
Psychosocial	71.78 (19.59)
PedsQL, Rheumatology Module, mean (S.D.) ^a^	
Pain and hurt	65.80 (25.94)
Daily activities	85.91 (19.77)
Treatment	69.51 (21.65)
Worry	67.17 (24.16)
Communication	64.51 (28.97)

^a^scale 0 – 100, higher score = better HRQoL

In univariate analysis, the independent variables that were associated with better Physical HRQoL were: male gender (t = 2.12, df = 114, *p* < 0.05); fewer active joints (r_s_ = −0.22, *p* < 0.05); greater perceived ability to take MTX (r = 0.38, *p* < 0.005) and greater perceived helpfulness of MTX (r = 0.30, *p* = 0.001). The independent variables that were associated with better Psychosocial HRQoL were: fewer active joints (r_s_ = −0.23, *p* < 0.05); oral administration of MTX (t = 2.27, df = 113, *p* < 0.05), greater perceived ability to take MTX (r = 0.38, *p* < 0.005) and greater perceived helpfulness of MTX (r = 0.27, *p* < 0.005).

Multivariate analyses of the relation between experiences of MTX and physical and psychosocial HRQoL are shown in Table 3. CYP’s perceptions of MTX made a small but statistically significant contribution to explaining variability in HRQoL. MTX-related variables explained an additional 9 % and 16 % respectively in physical and psychosocial HRQoL after controlling for gender and disease activity, as shown by the change in cumulative adjusted R^2^ in Table 3. After controlling for demographic and disease variables, poorer physical HRQoL was significantly accounted for by greater reported difficulty in taking MTX. Poorer psychosocial HRQoL was significantly accounted for by subcutaneous MTX administration, a lower rating of MTX helpfulness and a greater reported difficulty in taking MTX.

**Table 3:** Multiple regression analyses of variables related to health-related quality of life

	PedsQL Physical			PedsQL Psychosocial		
Variables	β	t	Cumulative Adjusted R^2^	β	t	Cumulative Adjusted R^2^
Demographics:			0.03			
Gender	0.149	−1.645		-	-	
Disease activity:			0.05			0.03
Active joints	0.110	−1.195		−0.126	−1.422	
MTX:			0.14			0.19
Subcutaneous route	-	-		−0.197	−2.216*	
PARQ Ability to take	0.256	2.688**		0.270	2.912**	
PARQ Helpfulness	0.159	1.732		0.217	2.408*	

*p < 0.05, **p < 0.01

## Discussion

This is the first study of which we are aware that has reported CYP’s views about taking MTX for JIA in relation to their HRQoL. In the multiple regression analyses MTX-related variables made an independent contribution to explaining variance in physical and psychosocial HRQoL after controlling for demographic and disease-related variables. Physical HRQoL was poorer in those who reported greater difficulty in taking MTX. Psychosocial HRQoL was poorer in those who: took MTX subcutaneously rather than orally; reported a greater level of difficulty in taking MTX and reported a lower level of helpfulness of MTX. Our findings concur with those of Seid et al. 2014 [7] and Haverman et al. 2012 [13], which found that self-reported problems with treatment were related to poorer HRQoL. The current study found that MTX-related factors were important in explaining both physical and psychological HRQoL as measured by the PedsQL.

We have previously reported findings from the mothers of CYP in the CHARMS study [9]. Approximately half of CYP were reported by their mothers to have experienced MTX side effects and/or procedural anxiety regarding injections or blood tests. The child assessment we report in this paper used a simpler, less detailed measure of MTX-related difficulties, so the results are not directly comparable but the finding of a relationship between problems taking MTX and poorer HRQoL is consistent across the respondents.

Receiving MTX to treat JIA has been shown to have a beneficial effect on CYP’s HRQoL [3], however those CYP who experience difficulty in taking MTX may not gain the full benefit. This study has shown that although most CYP rated MTX as helpful and reported high adherence, a significant minority report difficulties taking MTX and these difficulties were associated with poorer HRQoL. Clinicians who ask directly about CYPs’ experiences of taking MTX may be able to further enhance the HRQoL of their patients by offering treatments to help address these difficulties.

Psychosocial HRQoL was poorer in CYP taking MTX by subcutaneous rather than oral route. The data for this study were collected before the introduction of the Metoject pen. It would be of interest to examine whether use of the pen has an impact on pain and/or anxiety and any consequent impact on HRQoL.

The study has several limitations. As the study is cross-sectional, the direction of causation is unclear therefore it is possible that those children with poorer HRQoL have a generally more negative outlook and perceive MTX more negatively. We have however controlled for disease activity in the analysis (see Table 3) which indicates that experience of MTX is an independent predictor of HRQoL after taking disease activity into account i.e. it is not the case that the findings are explained simply by CYP with more active joints perceiving MTX more negatively.

The study reports HRQoL at a single time-point in those CYP currently taking MTX. Although the CHARMS study included CYP who were no longer taking MTX, the study was not examining reasons for stopping MTX and therefore this information was not recorded. It is possible that HRQoL would have varied in those CYP who stopped MTX due to intolerance or remission but we were unable to examine these differences. We therefore limited the analyses in this paper to CYP currently taking MTX. It would be informative to examine CYPs’ experiences of taking MTX and HRQoL over time from when they first take the medication.

As the study respondents are CYP, it is limited to those aged eight years and over. However, a strength of the CHARMS study is that we collected data from both parents and CYP so our related publication reporting mothers’ views was able to include proxy reports for younger children. A limitation of using a VAS to measure participants’ views about MTX is that it is not clear what cut-off scores on the 100 mm scales should be considered to signify, for example, mild, moderate and severe problems in taking MTX and therefore what percentage of CYP would fall into each category. The CYP in this study were already being treated with MTX for varying durations when they were recruited therefore it was not possible to control for level of response to MTX in our analysis. We did, however, include an indicator of disease severity in the number of active and limited joints.

## Conclusions

In conclusion, this analysis of CYP’s views about and experience of taking MTX supports the findings from our reports of mothers of CYP with JIA that MTX is viewed as helpful by most CYP but HRQoL is poorer in those who report greater difficulty in taking MTX.
